# Kidney length normative values in children aged 0–19 years — a multicenter study

**DOI:** 10.1007/s00467-021-05303-5

**Published:** 2021-10-16

**Authors:** Łukasz Obrycki, Jędrzej Sarnecki, Marianna Lichosik, Małgorzata Sopińska, Małgorzata Placzyńska, Małgorzata Stańczyk, Julia Mirecka, Agnieszka Wasilewska, Maciej Michalski, Weronika Lewandowska, Tadeusz Dereziński, Michał Pac, Natalia Szwarc, Karol Annusewicz, Viktoriia Rekuta, Karolis Ažukaitis, Andrius Čekuolis, Aldona Wierzbicka-Rucińska, Augustina Jankauskiene, Bolesław Kalicki, Katarzyna Jobs, Marcin Tkaczyk, Janusz Feber, Mieczysław Litwin

**Affiliations:** 1 Department of Nephrology, Kidney Transplantation and Hypertension, Children’s Memorial Health Institute, al. Dzieci Polskich 20, 04-730 Warsaw, Poland; 2 Department of Diagnostic Imaging, Children’s Memorial Health Institute, Warsaw, Poland; 3 Department of Paediatrics, Paediatric Nephrology and Allergology, The Military Institute of Medicine, Warsaw, Poland; 4 Department of Pediatrics, Immunology and Nephrology, Polish Mother’s Memorial Hospital Research Institute, Łódź, Poland; 5 Department of Radiology, Polish Mother’s Memorial Hospital Research Institute, Lodz, Poland; 6 Outpatient Clinic “Esculap”, Gniewkowo, Poland; 7 Department of Pediatrics, Jan Bogdanowicz Children’s Hospital, Warsaw, Poland; 8 Department of Paediatrics, Nutrition and Metabolic Diseases, Children’s Memorial Health Institute, Warsaw, Poland; 9 Clinic of Pediatrics, Faculty of Medicine, Vilnius University, Vilnius, Lithuania; 10 Department of Pediatric Radiology, Vilnius University Hospital Santaros Clinics, Vilnius, Lithuania; 11 Department of Biochemistry and Experimental Medicine, Children’s Memorial Health Institute, Warsaw, Poland; 12 Division of Nephrology, Department of Pediatrics, The Children’s Hospital of Eastern Ontario, Ottawa, Canada; 13 Chair of Pediatric Nephrology, Medical Center for Postgraduate Education, Warsaw, Poland

**Keywords:** Kidney, Kidney length, Children, Normal references, Ultrasonography

## Abstract

**Background:**

Currently used pediatric kidney length normative values are based on small single-center studies, do not include kidney function assessment, and focus mostly on newborns and infants. We aimed to develop ultrasound-based kidney length normative values derived from a large group of European Caucasian children with normal kidney function.

**Methods:**

Out of 1,782 children aged 0–19 years, 1,758 individuals with no present or past kidney disease and normal estimated glomerular filtration rate had sonographic assessment of kidney length. The results were correlated with anthropometric parameters and estimated glomerular filtration rate. Kidney length was correlated with age, height, body surface area, and body mass index. Height-related kidney length curves and table were generated using the LMS method. Multivariate regression analysis with collinearity checks was used to evaluate kidney length predictors.

**Results:**

There was no significant difference in kidney size in relation to height between boys and girls. We found significant (*p* < 0.001), but clinically unimportant (Cohen’s *D* effect size = 0.04 and 0.06) differences between prone vs. supine position (mean paired difference = 0.64 mm, 95% CI = 0.49–0.77) and left vs. right kidneys (mean paired difference = 1.03 mm, 95% CI = 0.83–1.21), respectively. For kidney length prediction, the highest coefficient correlation was observed with height (adjusted *R*^2^ = 0.87, *p* < 0.0001).

**Conclusions:**

We present height-related LMS-percentile curves and tables of kidney length which may serve as normative values for kidney length in children from birth to 19 years of age. The most significant predictor of kidney length was statural height.

**Graphic Abstract:**

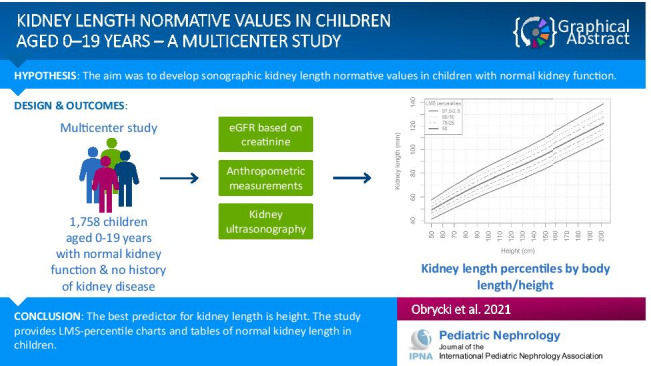

**Supplementary Information:**

The online version contains supplementary material available at 10.1007/s00467-021-05303-5.

## Introduction

The assessment of kidney size is of indisputable importance in the diagnostics of pediatric kidney diseases. First normative values of kidney size in children were published almost 60 years ago by Hodson et al., who assessed the kidney length based on radiographs (intravenous pyelograms) in 393 children aged 0–16 years [1]. Following the introduction of ultrasound examinations, Rosenbaum et al. published the first ultrasound-based normative data of kidney length in 1984, which many clinicians still use. These norms were based on 203 patients aged 0–19. Although the authors excluded patients with an obvious abnormality of the upper urinary tract (e.g., hydronephrosis or vesicoureteral reflux greater than grade 1), some of the study subjects had history/evidence of urinary tract infections or other urinary diseases, including abnormal urinalysis or enuresis, that could be associated with changes in the structure of the parenchyma, and therefore kidney length [2].

Until now, several other studies analyzing kidney length and volume in healthy children were published, but most of them were single-center studies on a relatively small number of patients, mainly focusing on newborns and infants rather than older children [3–17]. In addition, in none of the published studies so far was the kidney function assessed, which may have led to the inclusion of patients with undiagnosed kidney diseases.

Our multicenter study aims to develop ultrasound-based kidney length normative data in children and adolescents aged 0–19 years without any history of kidney disease and normal kidney function.

## Methods

We enrolled 1,782 Caucasian children recruited between 2018 and 2021 from Polish schools, kindergartens, sports clubs, and Polish and Lithuanian pediatric wards. All subjects were included in the study after a written consent signed by parents and/or children over 16 years. Invitations to participate in the study were sent out to random schools in Warsaw and Toruń (urban areas) and Inowrocław and Gniewkowo (suburban/rural areas). All consecutive patients admitted to inpatient clinics (Warsaw, Łódz, and Vilnius) in the study period were screened for inclusion in the study; those with non-nephrological and not chronic conditions, who signed informed consent, were subsequently included in the study.

The exclusion criteria were history of kidney disease (reported by parents or older subjects) or any other abnormalities of abdominal ultrasound, including ectopic kidney, kidney agenesis, horseshoe kidney, kidney duplication, tumors, cystic lesions, hydronephrosis (pelvic anterior–posterior diameter > 5 mm in neonates or > 10 mm in older participants), impaired kidney function (eGFR < 90 mL/min/1.73m^2^ in children > 1 year of age, eGFR < 60 mL/min/1.73m^2^ in children < 1 year of age), and any evidence of chronic disease (assessed by the physician at the time of the ultrasound exam). In addition, all study subjects had anthropometry and serum creatinine measured on the day of the ultrasound exam. The study was approved by the Bioethics Committee of the Children’s Memorial Health Institute and the Vilnius Regional Bioethics Committee. Furthermore, all patients and their parents gave informed consent. Thus, the study meets the criteria of the 1975 Declaration of Helsinki, revised in 2013.

### Sample size calculation

To ensure a 95% CI of the mean kidney length in children of each age category within 5 units of the true mean, a sample of at least 50 children per age category was needed.

### Anthropometric techniques

Height was measured in duplicate using a SECA 214 stadiometer (Seca GmbH & Co. KG, Hamburg, Germany). Each participant was in the upright standing position with shoes off, hips and shoulders perpendicular to the central axis, heels against the footboard, knees together, arms hanging loosely at the sides, and the head in the Frankfurt plane. In children in whom the standing height cannot be measured (usually younger than 2 years), the height was measured using the portable Harpenden Infantometer (range 30–110 cm). Height was recorded to the nearest 0.5 cm. The body weight of participants, who wore light underwear, was recorded to the nearest 0.5 kg, using a digital medical scale (Radwag WPT 100/200, Poland). Body mass index (BMI) was calculated as body weight divided by height in meters squared. Body surface area (BSA) was calculated using the Haycock formula [18]:

$$BSA[{m}^{2}]=0.024265\times {H}^{0.3964}[cm]\times {W}^{0.5378}[kg]$$

H — body length/height

W — body weight

### Laboratory measurements

Blood (2 mL) was drawn in the treatment room of the ward, school, or kindergarten after 12 h of fasting and was centrifuged at 1,465 × *g* at room temperature for 10 min. The serum creatinine concentration test was performed using the enzymatic method with commercial kits using an autoanalyzer A15 (BioSystems) [19]. The estimated glomerular filtration rate was calculated using the modified Schwartz formula [20]:

$$eGFR [mL/min/{1.73m}^{2}]=0.413\times H[cm]/SCr[mg/dL]$$

H — body length/height

SCr — serum creatinine concentration

We used the Schwartz formula which is currently considered the best method for estimating eGFR in children, known as the “Bedside Schwartz” formula. In most cases, the Schwartz equation allows for a rapid and reasonably accurate estimation of eGFR for clinical use in children with CKD and in accordance with the KDIGO guidelines.

### Kidney length assessment technique

Ultrasound examinations of kidney size were performed using Toshiba Aplio i700, Toshiba Aplio i800, Mindray Resona 7, Samsung RS80, and Philips Lumify units. The studies were conducted using curved array transducers by trained pediatric radiologists or pediatricians with at least 3 years of experience in sonography. Children were examined in both supine and prone positions, except for neonates in whom the measurements were made in supine and right and left lateral decubitus positions. After kidney identification, it was visualized along its longitudinal axis crossing through the kidney hilum. At least three measurements of maximal kidney length, from upper to lower pole, with an accuracy of 0.1 mm were performed in each body position. The maximal measured values of kidney length in millimeters for both supine and prone positions were recorded for each patient.

### Statistical analyses

All analyzed variables were checked for normality of distribution with the Shapiro–Wilk test. However, to compare normally and non-normally distributed variables across all age and height categories, all parameters are shown as median and interquartile range. The paired comparisons of left vs. right kidney size differences as well as kidney size differences measured in prone vs. supine position were carried out using distribution-free permutation tests with Cohen’s *D* effect size estimates and bias-corrected and accelerated confidence intervals (Python, dabest package). The relationships between the lateral/prone-supine differences in kidney length and age/height were analyzed using quantile regression (R, quantreg package). Differences in kidney length between boys and girls of the same age were analyzed using the Wilcoxon unpaired test. The relationship between kidney size and various anthropometric parameters (age, height, body surface area, body mass index) was assessed by non-parametric quantile regression (R, GAMLSS package). Height-related kidney length curves and tables were generated using the LMS method (R, GAMLSS package). Predictors of kidney length were evaluated using multivariate regression analysis with checks for collinearity using variation inflation factor and mixed linear model with age, sex, weight, height, and BMI (fixed effects), and age category and BMI within age category as random effects (R, packages ‘car’ and ‘lme4’). *P*-values < 0.05 were considered statistically significant. All analyses were performed using Python v.3.8 (Jupyter Lab) and R v.4.0.4 (RStudio v. 1.4.1106).

## Results

The final study group consisted of 1,758 children (aged 0–19 years, including 868 boys; 49%), as 24 individuals were excluded due to incomplete data, abnormal kidney function, or pathologies identified during sonographic examination. There were between 62 and 213 patients in each age category. Anthropometric and kidney function parameters (per age category) are shown in Table 1. All children included in the final analysis had normal kidney function (overall median = 112.7 mL/min/1.73 m^2^, IQR = 95.9 to 133.5), as per inclusion criteria. Median (interquartile range) BMI SDS of all children was 0.06 (− 0.78, 0.97); boys had a significantly higher median BMI SDS (+ 0.17; IQR =  − 0.67 to + 1.11) than girls (− 0.01; IQR =  − 0.88 to + 0.85) (*p* = 0.0012). A total of 225 children had BMI SDS > 1.65 (95th percentile), evenly distributed (5–15% of each category) across the whole age range except for age categories from 9 to 12 years, in which the incidence of obesity reached 20–30% (predominantly boys) (Table 1).

**Table 1:** Characteristics of the patient group

Age (years)	Number of patients	Weight [kg]	Body length/height [cm]	BSA [m^2^]	BMI [kg/m^2^]	BMI z-score	eGFR [mL/min/ 1.73m^2^]	Number of patients with BMI *z*-score > 1.65
0–1	213 (55% boys)	6.9 (5.2;8.5)	66 (60;71)	0.36 (0.3;0.42)	15.5 (14.1;17.0)	− 0.73 (− 1.67;0.24)	122.2 (103.3;140.4)	12 (6%)
1–2	101 (51% boys)	11.1 (10;12)	82 (79;86)	0.51 (0.3;0.42)	16.02 (15.1;17.3)	0.02 (− 0.72;0.82)	118.4 (110.1;152.8)	9 (9%)
2–3	70 (47% boys)	13 (12;14)	91 (88.3;97.8)	0.58 (0.55;0.61)	15.8 (14.8;17.3)	0.09 (− 0.74;1.15)	123.9 (116.0;140.4)	10 (14%)
3–4	77 (44% boys)	15.4 (14;17)	100.5 (97;104)	0.66 (0.62;0.70)	15.5 (14.6;16)	0.03 (− 0.57;0.5)	120.5 (103.3;138.5)	5 (6%)
4–5	71 (58% boys)	18 (16.9;19.5)	110 (106.5;112)	0.73 (0.71;0.78)	15.04 (14.0;16.05)	− 0.15 (− 0.97;0.58)	114.6 (109.7;143.5)	6 (8%)
5–6	81 (57% boys)	20.2 (20;25)	116 (111;119)	0.81 (0.75;0.87)	15.54 (14.4;17.0)	0.21 (− 0.74;1.08)	118.8 (100.8;137.0)	10 (12%)
6–7	73 (49% boys)	22 (20;25)	122 (118;128)	0.86 (0.81;0.94)	14.9 (13.9;16.4)	− 0.31 (− 1.26;0.69)	123.0 (102.2;122.9)	6 (8%)
7–8	115 (49% boys)	25 (22.7;30.3)	126 (122;130)	0.93 (0.87;1.04)	16.1 (14.8;17.8)	0.28 (− 0.58;1.2)	122.4 (93.5;142.0)	16 (14%)
8–9	84 (55% boys)	28.6 (26;32.1)	134 (130;138)	1.03 (0.96;1.1)	16.3 (15;18)	0.21 (− 0.63;1.05)	109.0 (90.4;130.3)	12 (14%)
9–10	83 (49% boys)	32 (28;37.5)	140 (135.8;143)	1.11 (1.01;1.23)	16.4 (14.9;18.7)	0.08 (− 0.81;1.17)	101.5 (85.5;124.0)	13 (16%)
10–11	102 (60% boys)	36 (30.5;43.4)	144.5 (139;148)	1.20 (1.08;1.35)	17.4 (15.7;20.2)	0.25 (− 0.64;1.3)	104.1 (89.7;123.7)	21 (21%)
11–12	112 (45% boys)	46.5 (37.8;54.2)	152 (147;158)	1.42 (1.24;1.54)	19.6 (16.9;22.8)	0.85 (− 0.33;1.78)	109.9 (86.7;135.4)	33 (29%)
12–13	108 (53% boys)	49.8 (41.8;59.2)	158.5 (152.9;164.6)	1.47 (1.34;1.64)	19.4 (17.3;23.1)	0.43 (− 0.28;1.67)	109.4 (91.5;122.8)	29 (27%)
13–14	96 (49% boys)	50.5 (45;57)	163 (155.5;169)	1.52 (1.40;1.63)	19.5 (17.8;21.5)	0.11 (− 0.47;0.84)	111.3 (92.2;137.8)	8 (8%)
14–15	76 (41% boys)	55 (50;60)	167.8 (162.8;173)	1.60 (1.51;1.68)	19.9 (18.21.7)	0.07 (− 0.7;0.64)	108.0 (93.3;132.5)	8 (11%)
15–16	73 (49% boys)	60 (50.8;67)	170 (163.5;177.7)	1.68 (1.59;1.78)	20.1 (18.6;22.3)	− 0.08 (− 0.78;0.66)	106.5 (95.0;121.2)	6 (8%)
16–17	70 (34% boys)	60.9 (54;68)	167.3 (163;173.4)	1.69 (1.58;1.78)	21 (19.9;23)	0.01 (− 0.38;0.71)	106.2 (95.7;117.2)	3 (4%)
17–18	91 (38% boys)	63 (55.3;74)	171 (164; 179)	1.76 (1.59;1.90)	21.5 (19.5;23.7)	0.09 (− 0.67;0.7)	100.0 (93.0;115.8)	10 (11%)
18–19	62 (40% boys)	66.2 (57.1;78)	169.3 (163.1;178)	1.75 (1.63;1.97)	22.8 (20.4;25.5)	0.4 (− 0.31;1.1)	100.8 (87.2;106.6)	8 (13%)

The mean paired difference between the right and left kidney length across all age categories was 1.03 mm (95% CI =  − 0.83 to − 1.21, permutation *p* < 0.001) with the Cohen’s *D* effect size of only 0.06 (95% CI = 0.05 to 0.07). In addition, there was no significant association between kidney size difference and age in quantile regression with a median slope of 0.05 and *r*^2^ = 0.004; the median (50th percentile), and the 2.5th and 97.5th percentiles of the intercept were − 1.42 mm, − 6.7 mm, and + 3.6 mm, respectively. There was also no relationship between kidney size difference (right vs. left kidney) and height; all quantile regression slopes were close to 0.0, and the 5th, 50th, and 95th quantile intercepts were − 4.9, − 1.0, and + 1.25 mm, respectively.

The mean paired difference in the kidney length between the measurements in the prone and supine position was − 0.64 mm (95% CI =  − 0.49 to − 0.77, permutation *p* < 0.001) with the Cohen’s *D* effect size of only 0.04 (95% CI = 0.03 to 0.05). There was also no relationship between kidney size difference (in prone vs. supine position) and age or height; the quantile regression slopes were all close to 0, and the 5th, 50th, and 95th quantile intercepts were − 3.9, − 0.3, and + 4 mm for age, and − 2.4, 0.2, + 4 mm for height.

**Fig. 1:**
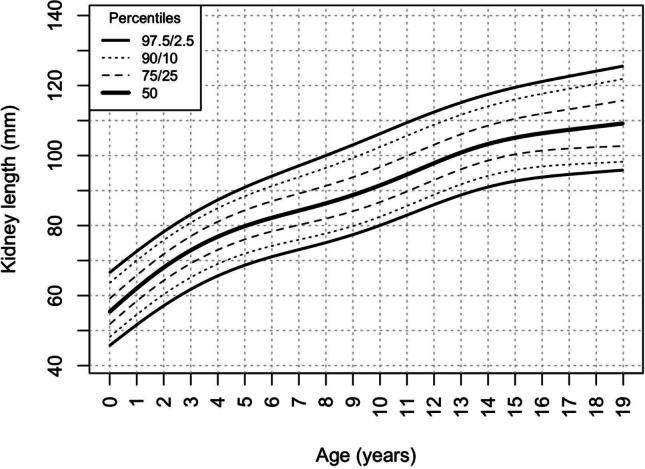
Kidney length percentiles by age

**Fig. 2:**
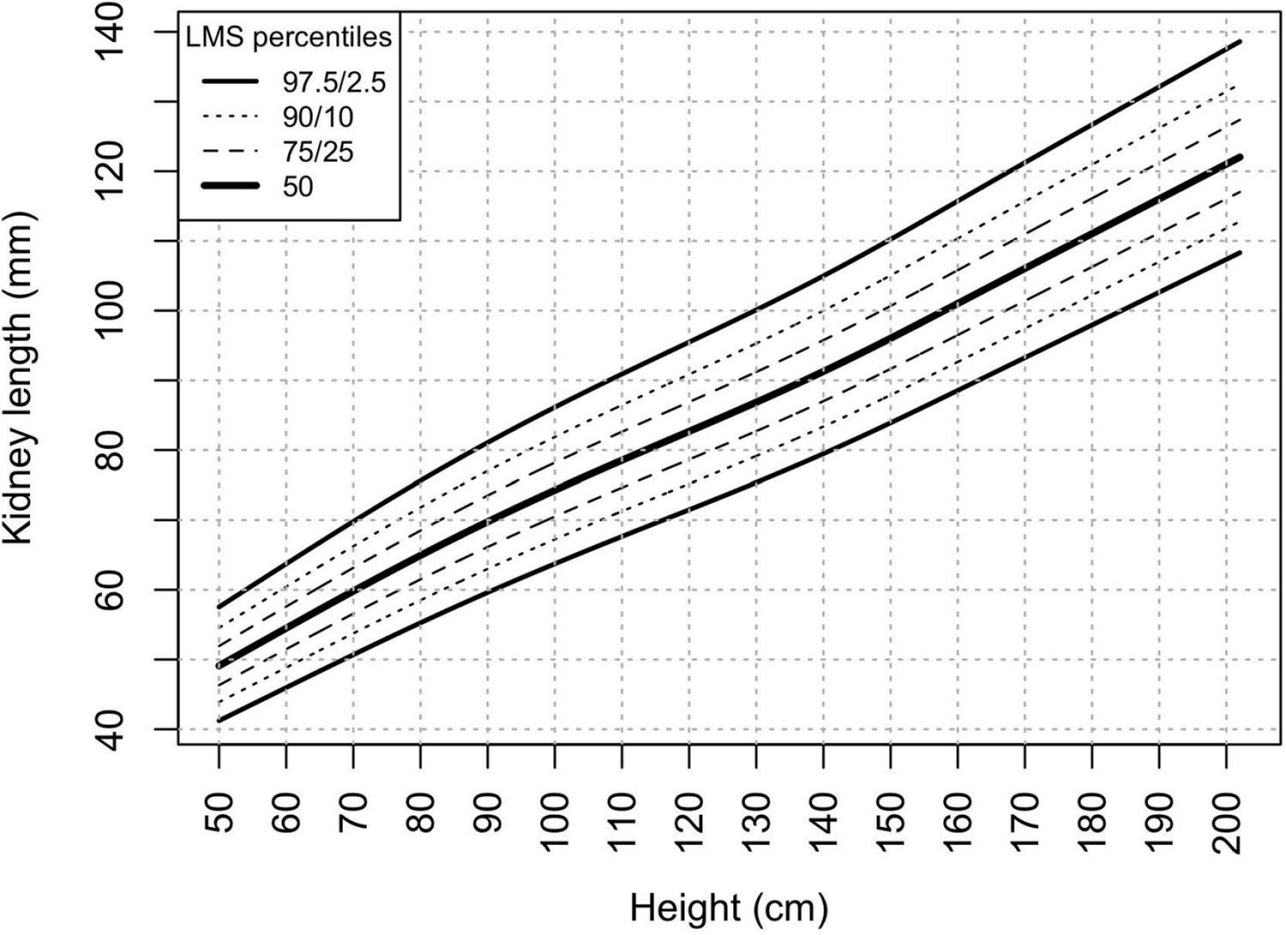
Kidney length percentiles by body length/height

**Fig. 3:**
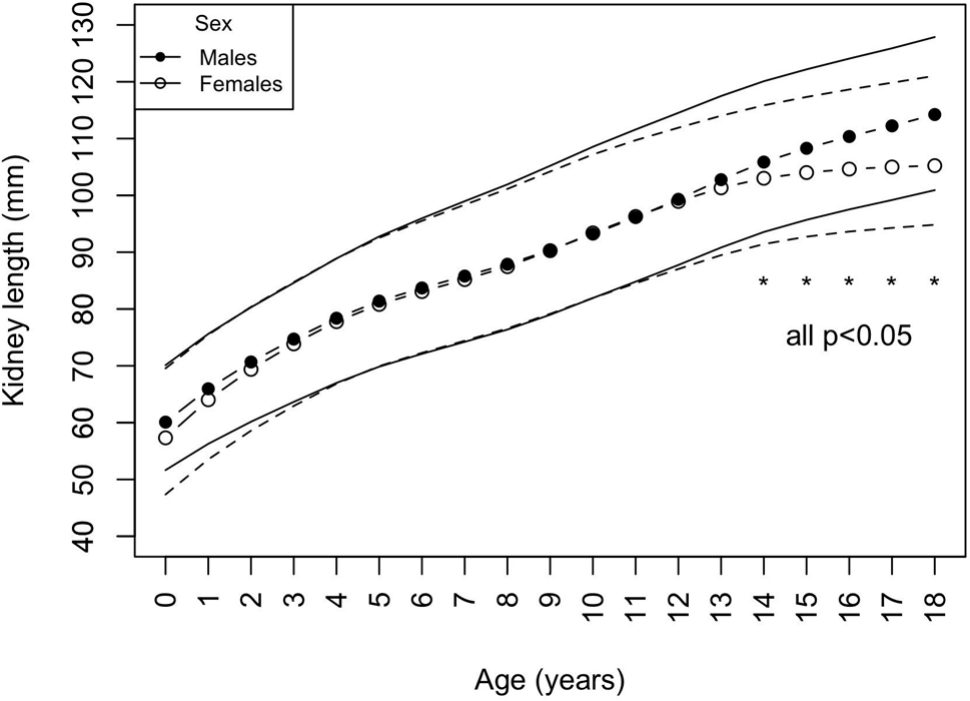
Kidney length by age and sex

The kidney length (median kidney length for each age and height category) increased gradually with age (from 60.1 mm in male infants to 114.2 mm in 18-year-old boys and from 57.3 mm in female infants to 105.2 mm in 18-year-old girls) (Table 2) (Fig. 1) and height (Fig. 2) (from 50.1 mm in newborns with a body length of 50–55 cm to 121.3 mm in adolescents with a height of 200–205 cm) (Table 3). There were no significant differences between kidney lengths in boys and girls in relation to height. However, boys aged 15 and higher had significantly larger kidneys than girls (Fig. 3), irrespective of their BMI.

**Table 2:** Kidney length percentiles by age

Age [years]	Sex	2.5th percentile [mm]	10th percentile [mm]	25th percentile [mm]	50th percentile [mm]	75th percentile [mm]	90th percentile [mm]	97.5th percentile [mm]
0–1	♂	51.6	53.6	56.8	60.1	63.3	67.4	70.1
	♀	47.4	49.7	53.4	57.3	62.3	66.9	69.5
1–2	♂	56.3	58.7	62.3	66.0	69.2	73.1	75.6
	♀	53.5	56.3	60.3	64.0	68.8	73.1	75.5
2–3	♂	60.2	62.9	66.9	70.7	74.1	77.9	80.3
	♀	58.6	61.6	65.7	69.4	74.0	78.1	80.4
3–4	♂	63.7	66.6	70.8	74.7	78.5	82.3	84.6
	♀	62.9	66.0	70.2	73.9	78.4	82.5	84.7
4–5	♂	67.0	70.1	74.4	78.4	82.6	86.5	88.9
	♀	66.8	69.9	74.1	77.8	82.2	86.5	88.9
5–6	♂	69.8	72.9	77.4	81.4	86.0	90.2	92.8
	♀	69.9	72.8	76.9	80.8	85.4	89.9	92.5
6–7	♂	72.1	75.1	79.6	83.7	88.6	93.1	96.0
	♀	72.3	74.9	79.0	83.1	87.8	92.6	95.5
7–8	♂	74.2	77.1	81.6	85.8	90.8	95.8	99.0
	♀	74.4	76.8	80.8	85.2	90.2	95.3	98.3
8–9	♂	76.4	79.1	83.5	87.9	93.0	98.4	101.9
	♀	76.6	78.8	82.9	87.4	92.8	98.0	101.1
9–10	♂	79.0	81.5	85.9	90.4	95.6	101.4	105.2
	♀	79.1	81.3	85.4	90.3	95.8	101.1	104.2
10–11	♂	81.9	84.4	88.6	93.2	98.5	104.6	108.6
	♀	81.9	84.2	88.4	93.4	99.0	104.3	107.2
11–12	♂	84.8	87.4	91.5	96.1	101.5	107.7	111.6
	♀	84.5	87.0	91.3	96.3	101.8	106.9	109.7
12–13	♂	87.7	90.4	94.5	99.3	104.6	110.7	114.5
	♀	87.0	89.7	94.0	98.9	104.3	109.1	111.9
13–14	♂	90.8	93.6	97.8	102.8	108.0	113.9	117.5
	♀	89.5	92.3	96.6	101.3	106.4	111.2	114.0
14–15	♂	93.6	96.4	100.8	105.9	111.0	116.7	120.1
	♀	91.4	94.2	98.4	103.0	108.0	112.9	115.8
15–16	♂	95.7	98.5	103.1	108.3	113.5	118.9	122.2
	♀	92.7	95.4	99.4	104.0	109.0	114.2	117.3
16–17	♂	97.5	100.3	105.0	110.4	115.7	121.0	124.1
	♀	93.6	96.0	99.9	104.6	109.8	115.4	118.6
17–18	♂	99.2	101.9	106.6	112.2	117.9	122.9	125.9
	♀	94.3	96.4	100.1	105.0	110.5	116.4	119.8
18–19	♂	100.9	103.5	108.3	114.2	120.2	125.0	127.9
	♀	94.8	96.6	100.0	105.2	111.4	117.5	121.0

**Table 3:** Kidney length percentiles by body length/height

Body length/height [cm]	2.5th percentile [mm]	10th percentile [mm]	25th percentile [mm]	50th percentile [mm]	75th percentile [mm]	90th percentile [mm]	97.5th percentile [mm]	M	L	S
50–54	42.4	45.1	47.6	50.4	53.3	56	59	50.4	0.567	0.0844
55–59	44.6	47.4	50	52.9	56	58.8	61.9	52.9	0.532	0.0836
60–64	46.8	49.7	52.4	55.4	58.6	61.5	64.8	55.4	0.498	0.0828
65–69	49.4	52.4	55.1	58.3	61.6	64.6	68.1	58.3	0.458	0.0820
70–74	51.6	54.7	57.5	60.8	64.2	67.3	70.9	60.8	0.423	0.0812
75–79	53.8	57	59.9	63.3	66.8	70	73.8	63.3	0.387	0.0804
80–84	55.9	59.2	62.2	65.7	69.3	72.6	76.5	65.7	0.352	0.0797
85–89	58.3	61.6	64.7	68.3	72	75.4	79.4	68.3	0.312	0.0788
90–94	60.3	63.7	66.9	70.6	74.3	77.9	82	70.6	0.276	0.0781
95–99	62.5	66	69.2	73	76.9	80.5	84.7	73	0.237	0.0773
100–104	64.5	68.1	71.4	75.2	79.1	82.8	87.1	75.2	0.2	0.0765
105–109	66.4	70	73.3	77.2	81.2	85	89.4	77.2	0.165	0.0758
110–114	68.2	71.8	75.2	79.1	83.2	87.1	91.6	79.1	0.131	0.0751
115–119	70.3	74	77.4	81.4	85.6	89.5	94.1	81.4	0.09	0.0743
120–124	72.3	76	79.5	83.5	87.8	91.8	96.4	83.5	0.052	0.0735
125–129	74.2	78	81.5	85.6	89.9	94	98.8	85.6	0.015	0.0728
130–134	76.2	80	83.6	87.8	92.2	96.3	101.1	87.8	− 0.022	0.0721
135–139	78.2	82	85.7	89.9	94.3	98.5	103.5	89.9	− 0.058	0.0714
140–144	80.3	84.2	87.8	92.1	96.6	100.9	105.9	92.1	− 0.093	0.0707
145–149	82.5	86.5	90.2	94.5	99.1	103.5	108.6	94.5	− 0.131	0.0700
150–154	84.8	88.8	92.6	97	101.7	106.1	111.3	97	− 0.168	0.0693
155–159	87.2	91.3	95.1	99.6	104.3	108.8	114.1	99.6	− 0.207	0.0686
160–164	89.5	93.6	97.5	102.1	106.9	111.5	116.9	102.1	− 0.243	0.0680
165–169	91.8	96	99.9	104.5	109.4	114	119.5	104.5	− 0.279	0.0673
170–174	94.1	98.3	102.3	106.9	111.9	116.6	122.2	106.9	− 0.315	0.0667
175–179	96.5	100.7	104.8	109.5	114.5	119.3	125	109.5	− 0.353	0.0660
180–184	98.8	103.1	107.1	111.9	117	121.9	127.6	111.9	− 0.389	0.0654
185–189	101.2	105.5	109.6	114.5	119.6	124.6	130.4	114.5	− 0.427	0.0647
190–194	103.3	107.7	111.9	116.8	122	127	132.9	116.8	− 0.461	0.0641
195–199	105	109.4	113.6	118.5	123.8	128.8	134.8	118.5	− 0.486	0.0637
≥ 200	108.3	112.8	117	122	127.4	132.5	138.6	122	− 0.538	0.0628

Table 3 shows height-stratified (by 5 cm categories) kidney length percentiles (2.5th, 10th, 50th, 75th, 90th, 97.5th percentiles) and LMS smoothing parameters for kidney length in relation to height. The LMS parameters allow for calculation of kidney length Z-scores (SDS) and/or percentiles according to the following formulas (and height-specific LMS parameters from Table 3):

$$Kidney\; length\; \text{Z-score}=((measured\ kidney\; length/M)^{L-1})/(L\times S)$$

$$Upper\ limit\ of\ kidney\ length\ (97.5^{th}\ percentile)\; [mm]:M\times(1+L\times S \times 1.96)^{1/L}$$

$$Lower\; limit\; of\; kidney\; length\; (2.5^{th}\; percentile)\; [mm]:M\times(1+L\times S \times-1.96) ^{1/L}$$

Simple formulas were developed (using quantile regression) to estimate the median (50th percentile) kidney length and the cut-off values for small (2.5th percentile) and enlarged kidney (97.5th percentile) using the patient’s height:

$$Median\; (50^{th}\; percentile)\; kidney\; length\; [mm]=0.5\times H[cm]+28.2$$

$$Upper\; limit\; of\; kidney\; length\; (97.5^{th}\; percentile)\; [mm]=0.5\times H[cm]+34.8$$

$$Lower\; limit\; of\; kidney\; length\; (2.5^{th} percentile)\; [mm]=0.4\times H[cm]+20.4$$

H — body length/height.

**Fig. 4:**
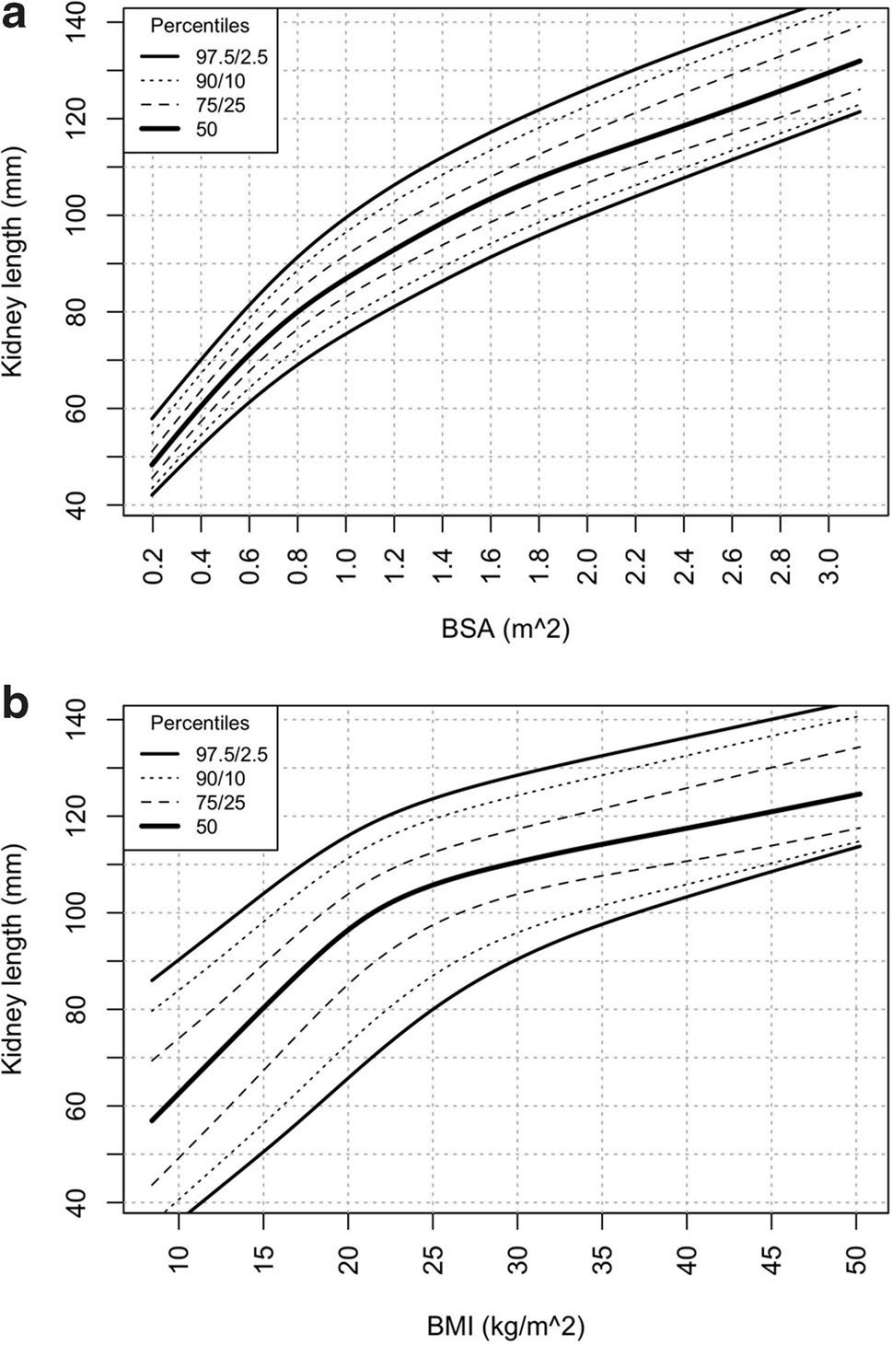
**a** Kidney length percentiles by BSA. **b** Kidney length percentiles by BMI

Figure 4a and b show the kidney length in relation to BSA (m^2^) and BMI (kg/m^2^), respectively. While there was a gradual increase in kidney length with increasing BSA (Fig. 4a), the increase of kidney length was steep up to BMI of 25, followed by a more gradual rise with BMI greater than 25 (Fig. 4b).

There was significant collinearity (variance inflation factor [VIF] ranging from 15 to 1395) between all independent variables/predictors, including age, height, BSA and BMI, and the log kidney length as the outcome variable, which limits the inclusion of all above mentioned predictors into the multiple regression analysis (in one formula). On regression analyses with log kidney length as dependent variable and independent predictors separately (age, height, BSA), the highest correlation coefficient was observed with height (adjusted *R*^2^ = 0.87, *p* < 0.0001), followed by BSA (adjusted *R*^2^ = 0.84, *p* < 0.0001) and age (adjusted *R*^2^ = 0.81, *p* < 0.0001). When BMI was added to the model as a second independent predictor (while keeping the VIF low, < 5), the relative importance of BMI was approximately 18.2% for the age model, 16.5% for the height model, and 19.1% for the BSA model. A combined linear mixed model with kidney length as outcome variable and age, sex, weight, height, and BMI as predictors (fixed effects) adjusted for age category (random intercept) and BMI within each age category (random slope) showed age, height, and BMI as significant predictors (*p*-values = 0.04, < 0.0001 and 0.01, respectively) of kidney length with *t*-values of 2.27 (95% CI = 0.02–0.48), 13.39 (95% CI = 0.32–0.45), and 2.99 (95% CI = 0.07–0.61), respectively. The age category accounted for 26% of total random effect variance, whereas the BMI had a negligible effect on random effects variance (< 1%).

**Fig. 5:**
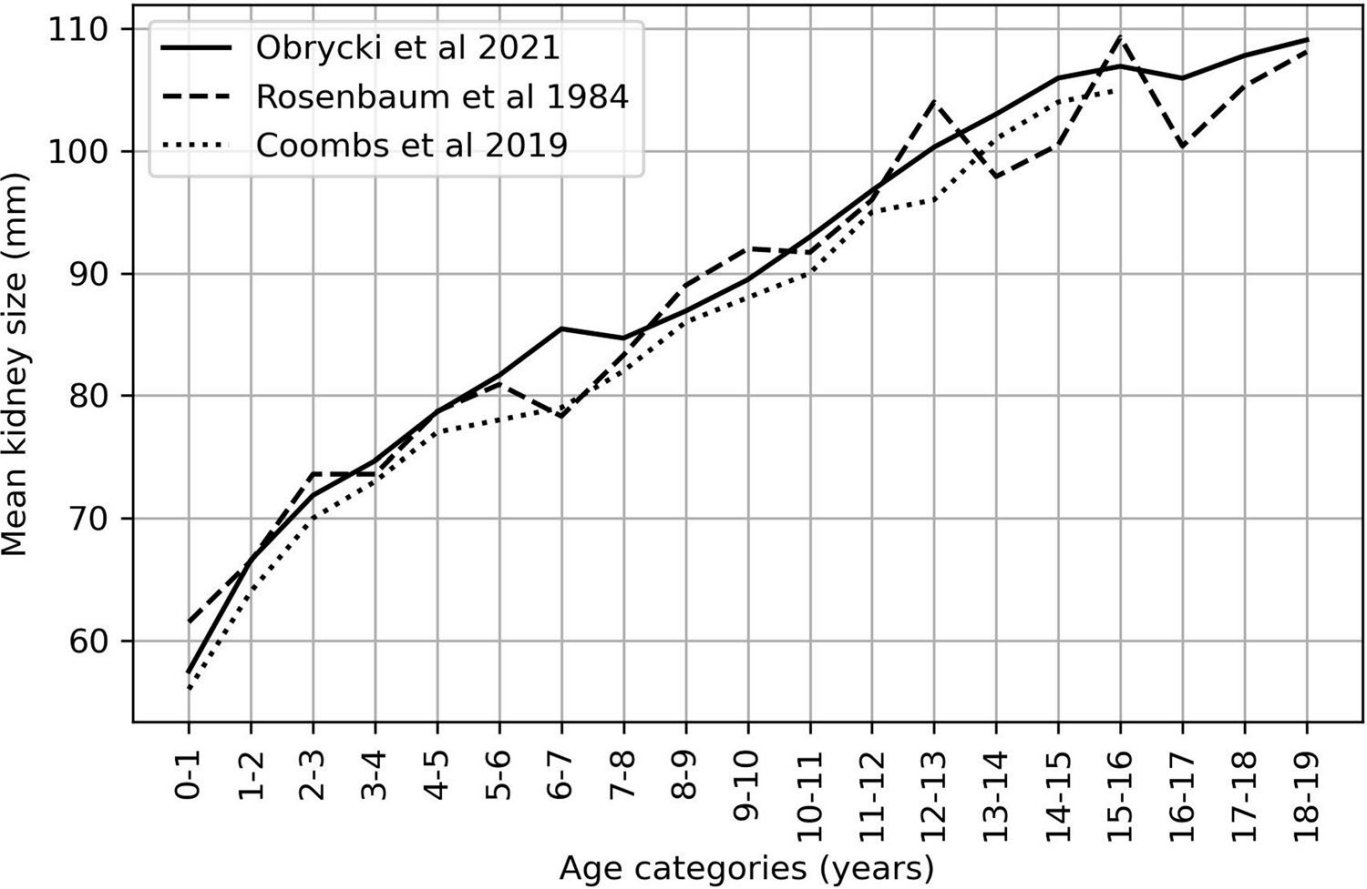
Comparison of different kidney length (median) normative values

We compared our normative data with reference values published by Rosenbaum et al. [2] and Coombs et al. [17]. As shown in Fig. 5, our age-related kidney length is more linear than that derived from Rosenbaum et al. Comparison of our age-related normative data with the study by Coombs et al. revealed higher values of kidney length in some age categories (from 0.9 mm in the 9th year of life to 6.4 mm in the 7th year of life).

## Discussion

We developed ultrasound-based normative values for kidney length in Central European children from birth to 19 years of age shown as age- and height-related percentiles, LMS-derived Z-scores, and quartile regression formulas. To our knowledge, our normative values are based on the largest group of children and adolescents published to date. Moreover, this is the first study on kidney length to include kidney function, which allowed us to exclude patients with impaired kidney function. This is also the first study on kidney length reporting LMS parameters for kidney size that can be used to calculate kidney length Z-scores and/or any given percentile of kidney length. For practical purposes, we developed percentiles (tables, curves) and simple formulas to calculate the 2.5th, 50th, and 97.5th percentiles of kidney size in relation to height.

### Boys/girls

Several previously published studies reported significant differences in kidney size between males and females. For example, in the longitudinal cohort study by Schmidt et al., boys had significantly larger kidney volumes than girls of all ages, and the sex difference was not due to body size [11]. Differences in kidney length between boys and girls were also described by Scott et al. on a group of 560 healthy infants [21]. However, the majority of studies reported no differences in kidney size between the sexes [1, 3, 7, 8, 15, 22]. We also did not observe significant differences in kidney length in relation to the height between girls and boys. However, boys over 15 years had significantly larger kidneys compared to girls of the same age (Fig. 3), which was most likely related to a pubertal growth spurt and greater height. This is similar to the differences in blood pressure values between the sexes. Furthermore, it is an additional argument for kidney size assessment based on height rather than age.

### Predictors of kidney length

Kidney size correlates well with most of the currently used parameters of body size, including height, weight, BMI, and BSA. According to a study by Dinkel et al. on 325 children aged between 3 days and 15 years, the best predictor of kidney length was BSA [5]. Similar results were obtained by Haugstvedt et al., who found a good correlation between kidney length and depth and variables like age, weight, height, and body surface area. However, BSA was the best predictor of kidney size [3].

Despite these significant correlations with BSA, it should be noted that the calculation of BSA is relatively cumbersome and requires measurements of both height and weight. In clinical practice, height and weight are measured directly in most patients. In contrast, BSA needs to be calculated, mostly for specific reasons only, e.g., as an index for GFR and left ventricular mass or drug dosing. In our study, there was also a significant correlation between BSA and kidney length (Fig. 4a); however, the correlation coefficient for BSA was still lower than the one for height.

While the relationship between age and kidney length is not linear (Fig. 1) and sex-dependent (adolescent boys have bigger kidneys than girls of the same age) (Table 2, Fig. 3), height was better correlated with kidney length with a higher r^2^ and no sex differences (Fig. 2).

Our findings are supported by the results of other studies in which height was the main predictor of kidney length. In the study published by Vujic et al., the strongest linear correlation coefficient was found between body length (height) and kidney length (*r* = 0.728 for the right kidney, *r* = 0.721 for the left kidney, and *r* = 0.724 for combined kidney length) and the combined kidney volume (*r* = 0.651) [12]. Similar findings were recorded by Thapa et al. on a group of 272 pediatric subjects aged between 1 month and 15 years; the kidney length showed the strongest correlation with height and age [16]. Height was also the main predictor of kidney length in children and adolescents in the study by Konus et al.; correlation coefficients with the kidney dimensions (longitudinal and transverse) were 0.94 and 0.86) [8]. In the study by Coombs et al., height and weight were not measured, and kidney length was related to age only [17]. Therefore, we conclude that from a statistical and clinical point of view, height seems to be the best predictor of kidney length irrespective of sex, age, and BMI. For clinical purposes, the median kidney length and its lower and upper limits (2.5th and 97.5th percentiles) can be predicted using simplified formulas (see above “Results”). For a more accurate assessment, the kidney length Z-scores/percentiles can be calculated using LMS parameters from Table 3.

The additional impact of BMI (besides age and height) on kidney length was further analyzed by multivariate analysis with BMI as a second independent predictor (in addition to age, height, or BSA). The relative impact of BMI on kidney length as an outcome measure was relatively small (up to 20%) compared to approximately 80% of variance accounted for by age, height, or BSA. The mixed linear model with age, sex, weight, height, and BMI showed that the most significant predictor of kidney length was height, followed by BMI and age (fixed effects), but the BMI had a negligible impact within age categories (random effect).

### Prone/supine position

The potential differences between kidney size in the prone and supine positions may be clinically important. Michel et al. found that the maximum measured longitudinal kidney length was statistically significantly larger in the supine than the prone position (supine position, left: 8.0 cm; right: 7.7 cm; prone position, left: 7.9 cm; right: 7.6 cm; *p* < 0.001). Therefore, the authors recommended including prone kidney length measurements in addition to the supine measurements. However, this would complicate follow-up examinations, as the kidney length measurements can only be compared with the previous measurement in the same patient position [23].

Our study also found a statistically significant difference in kidney size (mean paired difference =  − 0.64 mm, *p* < 0.001) between prone and supine positions. However, the absolute difference and effect size of this difference were minimal (Cohen’s *D* = 0.04), and the potential clinical significance may not be profound, especially given the existence of small intrinsic intra- and interobserver variability in sonographic length measurements [24, 25]. Therefore, we suggest measuring kidney length in any position in which kidney visualization is optimal.

### Left/right kidney

The differences in kidney length between the right and left kidney are another debated topic in the literature. In the study published by Blane et al. on 34 infants, the left kidney was found to be longer than the right one by 3 mm; however, the standard error of this prediction was 4.4 mm [4]. Scott et al. also confirmed that left kidneys were significantly longer and thinner than right ones. Although the differences in length and depth were highly significant, the confidence intervals showed that the scale of these differences was relatively small (about 1 mm) [21]. Left kidneys were also statistically significantly longer by approximately 2 mm in the normograms published by Haugstvedt et al. based on a group of 46 children aged 0–16 years [3]. Similar differences were observed in research by Kadioglu et al., including 292 children between 1 month and 18 years [13], and Michel et al., including 100 children from 6 months to 16 years [23]. In contrast, some other studies did not find significant lateral differences in kidney size [15, 17, 26]. Our study found a significant statistical difference between right and left kidney length (mean paired difference =  − 1.03 mm, *p* < 0.001). However, the absolute difference and its effect size were minimal (Cohen’s *D* = 0.06), not correlated with age or height. Therefore, we suggest that a statically significant lateral difference in kidney size is not clinically meaningful.

### Comparison with other normative data

The comparison between our age-related normative data and other studies published by Rosenbaum et al. and Coombs et al. is shown in Fig. 5 [2, 17]. While we could not compare the curves statistically (in the absence of raw data in other studies), the visual analysis of curves reveals some potential differences in age-related kidney length between studies. However, the age-related normative data are difficult to compare as they may be influenced by the abovementioned additional factors such as sex and BMI. Moreover, our study has the largest population studied so far (*n* = 1,758) compared to Rosenbaum et al. (*n* = 203) and Coombs et al. (*n* = 940). To our knowledge, our study is the first to include LMS smoothing parameters for kidney length (in relation to height) in children, which makes the kidney length assessment more precise. Therefore, we believe that our study provides accurate and up-to-date normative data for kidney length in children.

### Limitations

One of the limitations of our study was the lack of kidney volume assessment. Although kidney volume theoretically correlates better with kidney weight and would be preferable to know in certain conditions such as autosomal dominant polycystic kidney disease, the kidney length is directly measurable using abdominal ultrasonography. In contrast, the estimation of kidney volume in two-dimensional sonographic examination requires more measurements, which increases variability, and involves a relatively complicated calculation based on a geometric assumption about the shape of the kidney, which may be time-consuming in clinical practice [25, 27–29]. Therefore, most published normative data for kidney size rely on kidney length rather than volume. Another limitation may be the lack of intra- and inter-observer variability assessment of kidney length assessment in our study. However, we believe that the number of patients and a modern statistical approach (estimation-based analysis, including effect size analysis, quantile regression, LMS smoothing) significantly limits the variability and excludes extreme values/outliers.

## Conclusions

We found no clinically relevant sex- or patient position-related (supine or prone) differences in kidney size. The main determinant of kidney length was body height, which is also the most useful from the clinical perspective. Based on the largest pediatric cohort to date, our results can serve as reference values in clinical practice and research studies.

## Supplementary Information

Below is the link to the electronic supplementary material.

Supplementary file1 (PPTX 168 kb)

## Abbreviations

eGFR: Estimated glomerular filtration rate
BSA: Body surface area
BMI: Body mass index
GFR: Glomerular filtration rate
