# Supplementary material for: Kidney length normative values in children aged 0–19 years — a multicenter study
Source: Pediatr Nephrol. 2021 Oct 16;37(5):1075–85. doi: 10.1007/s00467-021-05303-5 (PMC9023417; doi:10.1007/s00467-021-05303-5)
Supplement: Supplementary file 1 — Supplementary file1 (PPTX 168 kb) [file 467_2021_5303_MOESM1_ESM.pptx]

## Slide 1
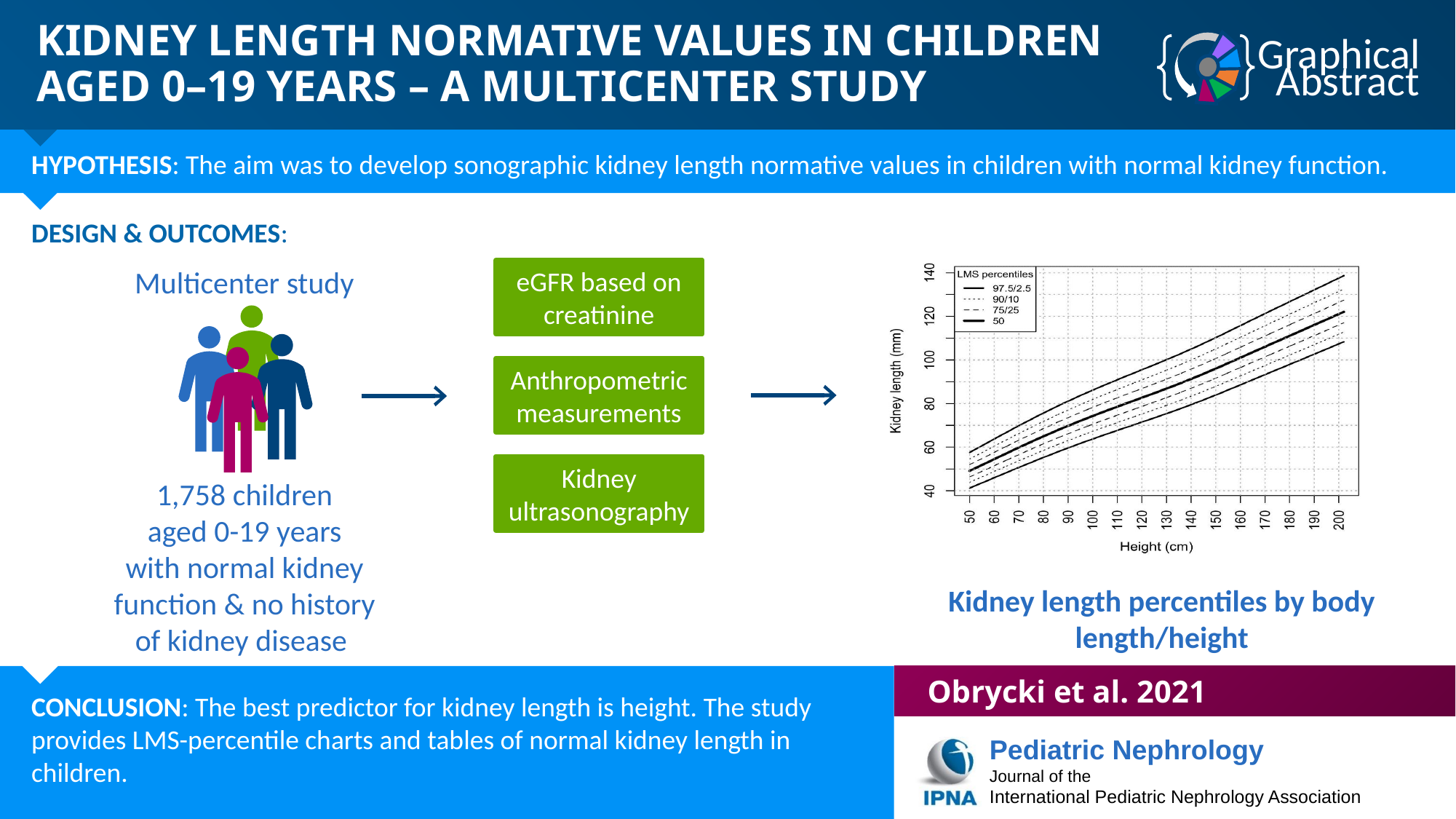

KIDNEY LENGTH NORMATIVE VALUES IN CHILDREN
AGED 0–19 YEARS – A MULTICENTER STUDY
HYPOTHESIS: The aim was to develop sonographic kidney length normative values in children with normal kidney function.
DESIGN & OUTCOMES:
Multicenter study
eGFR based on creatinine
Anthropometric measurements
Kidney ultrasonography
1,758 children
aged 0-19 years
with normal kidney function & no history of kidney disease
Kidney length percentiles by body length/height
Obrycki et al. 2021
CONCLUSION: The best predictor for kidney length is height. The study provides LMS-percentile charts and tables of normal kidney length in children.
